# Insulin-like growth factor-binding protein 7 alters the sensitivity to interferon-based anticancer therapy in hepatocellular carcinoma cells

**DOI:** 10.1038/sj.bjc.6605669

**Published:** 2010-04-20

**Authors:** Y Tomimaru, H Eguchi, H Wada, T Noda, M Murakami, S Kobayashi, S Marubashi, Y Takeda, M Tanemura, K Umeshita, Y Doki, M Mori, H Nagano

**Affiliations:** 1 Department of Surgery, Graduate School of Medicine, Osaka University, 2-2 Yamadaoka E-2, Suita, Osaka 565-0871, Japan; 2 Division of Health Sciences, Graduate School of Medicine, Osaka University, 2-2 Yamadaoka E-2, Suita, Osaka 565-0871, Japan

**Keywords:** hepatocellular carcinoma, interferon-*α*, 5-fluorouracil, insulin-like growth factor-binding protein 7 (IGFBP7)

## Abstract

**Background:**

A striking efficiency of interferon (IFN)-based anticancer therapy for advanced hepatocellular carcinoma (HCC) has been reported. Because its clinical efficiency greatly depends on each patient's local response, prediction of local response is crucial.

**Methods:**

Continuous exposure of IFN-*α* to parental PLC/PRF/5 cells (PLC-P) and a limiting dilution method resulted in the establishment of IFN-resistant cell clones (PLC-Rs). Microarray analyses of PLC-P and PLC-Rs identified insulin-like growth factor-binding protein 7 (IGFBP7) as one of the most significantly downregulated genes in PLC-Rs. Changes in anticancer effects of IFN-*α* were examined in HCC cells after genetic manipulation of IGFBP7 expression. The correlation between immunohistochemically determined IGFBP7 expression and the response to IFN-*α*/5-fluorouracil (5-FU) therapy was investigated in surgically resected HCC specimens.

**Results:**

PLC-R cells showed a remarkable downregulation of IGFBP7 and resistance to IFN-*α*, compared with PLC-P. Parental PLC/PRF/5 cells transfected with short hairpin RNA against IGFBP7 showed a significant resistance to IFN-*α* relative to control cells (IC_50_ fold increase=14.38 times). Insulin-like growth factor-binding protein 7 transfection into PLC-R restored sensitivity to IFN-*α*. In resected specimens, IGFBP7 expression significantly correlated with the response to IFN-*α*/5-FU therapy.

**Conclusion:**

IGFBP7 could be a useful predictor of the response to IFN-based therapy in advanced HCC.

The prognosis of patients with advanced hepatocellular carcinoma (HCC) remains poor, particularly in patients with tumour thrombi in the major trunk of the portal vein, even after curative resection of the tumour (Tanaka *et al*, 1996; Yamakado *et al*, 1999; Asahara, 1999 no. 47). In such a situation, conventional therapies have no clinical impact because of poor efficacy and possible complications (Furuse *et al*, 1997; Lee *et al*, 1997). Therefore, a new strategy is required for patients with advanced HCC.

Several studies have reported strong antitumour activity of interferon (IFN)-based combination chemotherapy to HCC, irrespective of the lack of satisfactory results of IFN-*α* monotherapy (Urabe *et al*, 1998; Leung *et al*, 1999; Chung *et al*, 2000; Patt *et al*, 2003; Obi *et al*, 2006). We have also reported the clinical efficiency of IFN-*α* and 5-fluorouracil (5-FU) (IFN-*α*/5-FU) therapy for advanced HCC and the underlying mechanisms of antitumour effects (Eguchi *et al*, 2000; Kondo *et al*, 2000, 2005; Sakon *et al*, 2002; Yamamoto *et al*, 2004; Ota *et al*, 2005; Nakamura *et al*, 2007; Wada *et al*, 2007, 2009; Damdinsuren *et al*, 2007a, 2007b; Nagano *et al*, 2007a, 2007b). These previous studies showed that IFN-*α* suppresses the proliferation of HCC cells that express type I IFN receptor type 2 (IFNAR2), and that the expression of IFNAR2 in HCC tissues was significantly associated with a clinical response to IFN-*α*/5-FU therapy, suggesting that IFNAR2 expression might be useful in predicting the clinical response to such therapy (Ota *et al*, 2005; Nagano *et al*, 2007a). However, even a portion of patients expressing IFNAR2 showed resistance to the therapy, indicating the necessity of finding novel biological markers that can more accurately predict the clinical response to IFN-*α*/5-FU therapy. Because the clinical outcome between responders and non-responders is markedly different, and to avoid the potentially debilitating adverse effects of this therapy in non-responders, finding the predictive biomarker is crucial.

In this study, IFN-resistant HCC cell clones were established and an oligonucleotide microarray analysis was applied to these IFN-resistant cells and their parental cells. The microarray analysis identified that insulin-like growth factor (IGF)-binding protein 7 (IGFBP7), which has been reported to have a tumour-suppressive activity through the induction of apoptosis in some cancers, was a key gene related to the response to this therapy (Burger *et al*, 1998; Landberg *et al*, 2001; Mutaguchi *et al*, 2003; Sato *et al*, 2007; Lin *et al*, 2008; Wajapeyee *et al*, 2008). Furthermore, we confirmed that IGFBP7 significantly correlated with the response to IFN-*α*/5-FU therapy in genetic manipulation experiments and to the clinical response in HCC tissue samples. These results indicate that IGFBP7 could be a suitable marker for predicting the clinical response to IFN-*α*/5-FU therapy.

## Materials and methods

### Cell lines

Human HCC cell lines, PLC/PRF/5 and HLE, were obtained from the Japan Cancer Research Resources Bank (Tokyo, Japan), and Hep3B was obtained from the Institute of Development, Aging and Cancer, Tohoku University (Sendai, Japan). These cells were maintained in Dulbecco's modified Eagle's medium supplemented with 10% fetal bovine serum, 100 U ml^−1^ penicillin and 100 mg ml^−1^ streptomycin at 37°C in a humidified incubator with 5% CO_2_ in air.

### Establishment of IFN-resistant cells

Parental PLC/PRF/5 cells (PLC-P) were exposed to IFN-*α* at an initial concentration of 50 IU ml^−1^. At 2 weeks after exposure, surviving cells were continuously exposed to sequentially increasing doses of 100 IU ml^−1^ (2 weeks), 200 IU ml^−1^ (2 weeks), 500 IU ml^−1^ (2 weeks), 1000 IU ml^−1^ (2 weeks), and 2000 IU ml^−1^. Through this process, we successfully established IFN-resistant cells. By limiting the dilution of the established cells, 10 clones of PLC/PRF/5 cells resistant to IFN-*α* were established. The clones were confirmed as being resistant to IFN-*α* stably over 20 passages. Among the 10 clones, three clones (PLC-Rs; PLC-R1, PLC-R2, and PLC-R3) were used in the experiments of this study.

### Drugs and reagents

Purified human IFN-*α* was kindly supplied by Otsuka Pharmaceutical Co. (Tokyo, Japan) and 5-FU and doxorubicin (DXR) by Kyowa Hakko Kirin Co. (Tokyo, Japan). Cisplatin (CDDP), insulin, and IGF-1 were purchased from Nippon Kayaku Co. (Tokyo, Japan), Sigma-Aldrich Co. (St Louis, MO, USA), and Peprotech (Rocky Hill, NJ, USA), respectively. As for primary antibodies, polyclonal goat anti-human IGFBP7 antibody and polyclonal rabbit anti-human IGFBP7 antibody (Santa Cruz Biotechnology Inc, Santa Cruz, CA, USA) were used for immunohistochemistry and western blot analysis, respectively. Antibodies to IFNAR2 and phosphotyrosine (p-Tyr) were purchased from Santa Cruz Biotechnology Inc; antibodies to signal transducer and activator of transcription factor (STAT) 1, phosphorylated (Tyr 701) STAT (pSTAT) 1, Akt, and phosphorylated (Ser 473) Akt were from Cell Signaling Technology (Beverly, MA, USA); antibodies to STAT2, pSTAT2, and insulin receptor substrate-1 (IRS-1) were from Millipore (Milford, MA, USA); and antibody to actin was from Sigma-Aldrich Co.

### Plasmid and transfection

Plasmid coding for short hairpin RNA (shRNA) against *IGFBP7* and *IGFBP7* expression plasmids was purchased from OriGene Technologies Inc. (Rockville, MD, USA). They were transfected into HCC cells using Lipofectamine 2000 (Invitrogen, Carlsbad, CA, USA) according to the instructions provided by the manufacturer. After transfection of the shRNA plasmid and *IGFBP7* expression plasmid, stable transfectants were selected and maintained by adding 1.0 *μ*g ml^−1^ of puromycin (Sigma-Aldrich Co.) and 600 *μ*g ml^−1^ of G418 (Gibco-BRL, Grand Island, NY, USA), respectively. The control vector plasmid expressing non-effective shRNA was similarly introduced into cells to establish negative control cells for the shRNA plasmid. Empty vector plasmid was also similarly used to establish negative control cells for the *IGFBP7* expression plasmid. Successful transfection was confirmed by the coexpression of GFP.

PLC-P transfected by shRNA plasmid against *IGFBP7* and by the negative control vector plasmid was named as PLC-P/shRNA (no. 1 and no. 2) and PLC-P/shRNA-NC, respectively. Short hairpin RNA no. 1 and no. 2 were different in sequence to shRNA. The PLC-Rs transfected with the *IGFBP7* expression plasmid and the negative control vector plasmid were named PLC-Rs/IGFBP7 and PLC-Rs/IGFBP7-NC, respectively.

### Patients and specimens

The study subjects were 30 patients with advanced HCC and recruited as described previously (Nagano *et al*, 2007a). All patients had multiple liver tumours in both lobes and tumour thrombi in the main trunk of the portal vein, and each underwent palliative reduction surgery with tumour thrombectomy of the main trunk of the portal vein at the Osaka University Hospital between October 1999 and December 2004. The IFN-*α*/5-FU therapy for remnant multiple liver tumours was applied postoperatively, as described previously (Ota *et al*, 2005; Nagano *et al*, 2007a). Patients were followed up after surgery, with a postoperative follow-up period of 18.2±19.7 months. Clinical response to therapy was evaluated according to the criteria of the Eastern Cooperative Oncology Group (Oken *et al*, 1982). On the basis of the clinical response, responders were defined as patients with a complete response or partial response and non-responders were defined as patients with a stable disease or progressive disease. The study protocol was approved by the Human Ethics Review Committee of Osaka University Hospital and a signed consent form was obtained from each patient.

### Real-time quantitative reverse transcription-PCR

For reverse transcriptase reaction, the extracted RNA, random hexamers, and Superscript II reverse transcriptase (Invitrogen) were used according to the instructions supplied by the manufacturer. Real-time quantitative reverse transcription-PCR (qRT-PCR) was performed using designed oligonucleotide primers and Light Cycler (Roche Diagnostics, Mannheim, Germany), and the amount of target gene expression was calculated. The expression of the target gene was normalised relative to the expression of *porphobilinogen deaminase* (*PBGD*), which was used as an internal control. The designed PCR primers were as follows: *IGFBP7* forward primer 5′-CTGGGTGCTGGTATCTCCTC-3′, *IGFBP7* reverse primer 5′-TATAGCTCGGCACCTTCACC-3′ *PBGD* forward primer 5′-TGTCTGGTAACGGCAATGCGGCTGCAAC-3′, *PBGD* reverse primer 5′-TCAATGTTGCCACCACACTGTCCGTCT-3′.

### Microarray experiments

Microarray experiments were conducted according to the method described previously (Noda *et al*, 2009). In brief, total RNA was purified by TRIzol reagent (Invitrogen) according to the instructions provided by the manufacturer. The integrity of the purified RNA was assessed as being of high quality by Agilent 2100 Bioanalyzer (Agilent, Santa Clara, CA, USA) and RNA 6000 LabChip kits (Yokokawa Analytical Systems, Tokyo, Japan). The purified RNAs obtained from PLC-P, PLC-R1, PLC-R2, and PLC-R3 were used as samples, and all samples were examined in duplicate. The samples were mixed and hybridised on a microarray covering 30 336 human probes (AceGene Human 30 K; DNA Chip Research Inc and Hitachi Software Engineering Co, Kanagawa, Japan). The ratio of the expression level of each gene was converted into a logarithmic scale (base 2) and the data matrix was normalised. In each sample, genes with missing values in more than two samples were excluded from the analysis. A total of 28 761 genes out of 30 336 genes were finally available for the analysis.

### Western blot analysis

Cells grown to semiconfluence were lysed in RIPA buffer (25 mM Tris (pH 7.5), 50 mM NaCl, 0.5% sodium deoxycholate, 2% Nonidet P-40, 0.2% sodium dodecyl sulphate, 1 mM phenylmethylsulphonyl fluoride, 1.6 *μ*g ml^−1^ aprotinin). Western blot analysis was carried out as described previously (Kondo *et al*, 2005).

### Growth inhibitory assay

The growth inhibitory assay was assessed by the 3-(4-,5-dimethylthiazol-2-yl)-2,5-diphenyl tetrazolium bromide (MTT) (Sigma-Aldrich Co.) assay, as described previously (Eguchi *et al*, 2000). Briefly, cells were incubated for 72 h under several concentrations of IFN-*α* and 5-FU. After reincubation for 4 h with MTT solution, acid–isopropanol mixture was added to dissolve the resultant formazan crystals. The absorbance of the plate was measured in a microplate reader at a wavelength of 570 nm with a 650 nm reference, and the results were expressed as a percentage of absorbance relative to that of untreated controls.

### Annexin V assay

The binding of annexin V was used as a sensitive method for measuring apoptosis, as described previously (Nakamura *et al*, 2007). At 24 h after treatment with IFN-*α*, PLC-P/shRNA and PLC-P/shRNA-NC cells were stained by Annexin V-APC and propidium iodide (PI) (BD Biosciences, Franklin Lakes, NJ, USA), and analysed on a FACS Aria (BD Biosciences). Annexin V-positive and PI-negative cells, considered as early apoptotic cells, were used for the assessment of apoptosis in this study (Lugli *et al*, 2005).

### Measurement of caspase activities

Caspase-3, caspase-8, and caspase-9 activities were measured using caspase-3, caspase-8, and caspase-9 colorimetric assay kits (Chemicon International Inc, Temecula, CA, USA). The measurement was performed in cell lysates obtained from each cell 24 h after treatment with IFN-*α*, using the instructions provided by the manufacturer.

### IRS-1 immunoprecipitation

After incubation for 12 h in serum-free medium, cells were stimulated with 1 nM insulin or 10 nM IGF-1. The stimulated cells were lysed in lysis buffer (20 mM Tris (pH 7.4), 150 mM NaCl, 1.0% Triton-X-100, 1.0 mM EGTA, 1 mM phenylmethylsulphonyl fluoride, 1.6 *μ*g ml^−1^ aprotinin, 10 *μ*g ml^−1^ leupeptin). Solubilised proteins were immunoprecipitated with anti-IRS-1 antibody, and tyrosine phosphorylation was detected with anti-p-Tyr antibody.

### Immunohistochemical staining

Immunohistochemical staining for IGFBP7 in 30 HCC samples was performed by the method described previously (Kondo *et al*, 1999). Briefly, formalin-fixed, paraffin-embedded 4 *μ*m-thick sections were deparaffinised in xylene, then treated with an antigen retrieval procedure and incubated in methanol containing 0.3% hydrogen peroxide to block endogenous peroxidase. After incubation with normal protein block serum, the sections were incubated overnight at 4°C with an anti-IGFBP7 antibody as the primary antibody. Thereafter, the sections were incubated with a biotin-conjugated secondary antibody (horse anti-goat antibody for IGFBP7) and with peroxidase-conjugated streptavidin. The peroxidase reaction was then developed with 0.02% 3, 30-diaminobenzidine tetrachloride (Wako Pure Chemicals, Osaka, Japan) solution with 0.03% hydrogen peroxide. Finally, the sections were counterstained with Meyer's haematoxylin. The IGFBP7 expression was defined as the presence of specific staining in the cytoplasm of cancer cells. Insulin-like growth factor-binding protein 7 expression was evaluated as positive or negative. Two investigators (Y.T. and H.E.) independently assessed IGFBP7 expression without knowledge of the corresponding clinicopathological data. The assessments were similar by the two investigators for all samples.

### Statistical analysis

Data are expressed as mean±s.d. Clinicopathological parameters were compared using the *χ*^2^-test and continuous variables were compared using Student's *t*-test. Survival curves were computed using the Kaplan–Meier method, and differences between survival curves were compared using the log-rank test. A *P*-value <0.05 denoted the presence of a statistically significant difference. Statistical analysis was performed using StatView (version 5.0, SAS Institute Inc, Cary, NC, USA).

## Results

### Characteristics of established IFN-resistant cells

**Figure 1:**
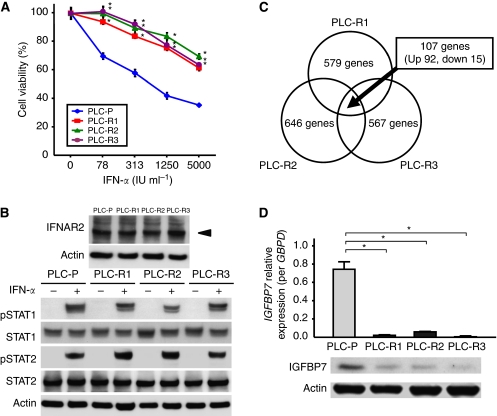
Characteristics of IFN-resistant PLC/PRF/5 cell clones (PLC-Rs). (**A**) MTT assay showed that the antitumour effect of interferon-*α* (IFN-*α*) in PLC-Rs was significantly lower than that in parental PLC/PRF/5 cells (PLC-P). Data are mean±s.d. ^*^P<0.05 compared with PLC-P. (**B**) Western blot analysis revealed that the expression levels of type I IFN receptor type 2 (IFNAR2), signal transducer and activator of transcription factor 1 (STAT1), STAT2, phosphorylated STAT1 (pSTAT1), and pSTAT2 were similar in PLC-P and PLC-Rs. (**C**) Schematic of the results of the performed microarray analysis and identified 107 genes. The 107 genes were up- or downregulated by more than 1.5-fold and were commonly identified in the three types of cells. (**D**) Quantitative reverse transcriptase-PCR and western blot analysis confirmed the significant suppression of insulin-like growth factor-binding protein 7 (IGFBP7) expression in PLC-Rs compared with PLC-P. Data are mean±s.d. ^*^P<0.05.

The morphology of PLC-Rs resembled that of PLC-P. Although PLC-Rs showed similar growth curves compared with PLC-P in the absence of IFN-*α* (data not shown), PLC-Rs were significantly resistant to IFN-*α* compared with PLC-P, which was confirmed by MTT assays (Figure 1A). The expression levels of IFNAR2 were not different between PLC-P and PLC-Rs (Figure 1B). The protein level of STAT1 and STAT2, which directly bind to the intracellular domain of IFNAR2 and function as key molecules for signal transduction, was also not different between PLC-P and PLC-Rs treated with 1000 IU ml^−1^ of IFN-*α* for 20 min (Figure 1B). Moreover, the phosphorylation of STAT1 and STAT2 (pSTAT1 and pSTAT2), active forms of STATs, were also not different between these cells.

### *IGFBP7* is significantly downregulated in IFN-resistant cells

To investigate the candidate genes involved in the response to IFN-*α*, microarray analysis was carried out with PLC-P and PLC-Rs. The analysis showed that, among the 28,761 genes, 579 (2.0%), 646 (2.2%), and 567 genes (2.0%) altered more than 1.5-fold in PLC-R1, PLC-R2, and PLC-R3, respectively. As shown in Figure 1C, 107 genes including 92 upregulated genes and 15 downregulated genes (listed in Supplementary Table S1) were common among the above 579, 646, and 567 genes. Among these 107 genes, *IGFBP7* was identified as one of the most downregulated genes with a 2.963-fold decrease. The downregulation of IGFBP7 in PLC-Rs compared with PLC-P was validated by real-time qRT-PCR and western blot analysis (Figure 1D).

### Knockdown of *IGFBP7* induces resistance to IFN-*α*

**Figure 2:**
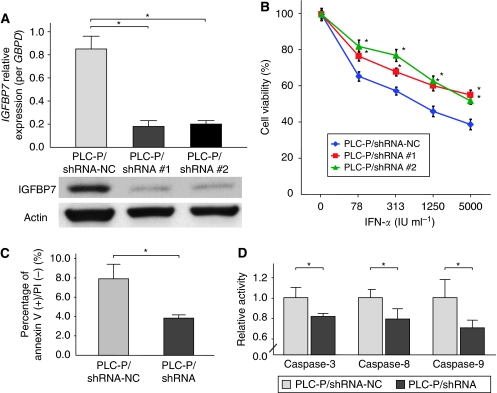
Characteristics of parental PLC/PRF/5 cell (PLC-P)/short hairpin RNA (shRNA) (no. 1 and no. 2). (**A**) Insulin-like growth factor-binding protein 7 (IGFBP7) was confirmed to be significantly suppressed in PLC-P/shRNA compared with PLC-P/shRNA-negative control (NC) in quantitative reverse transcriptase-PCR and western blot analysis. (**B**) MTT assay revealed that PLC-P/shRNA was significantly more resistant to interferon-α (IFN-*α*) than was PLC-P/shRNA-NC. (**C**) The percentage of early apoptotic PLC-P/shRNA cells assessed by annexin V assay was significantly lower than that of PLC-P/shRNA-NC. (**D**) The activity of caspase-3, caspase-8, and caspase-9 induced by IFN-*α* in PLC-P/shRNA was significantly lower than that in PLC-P/shRNA-NC. Data are mean±s.d. ^*^P<0.05.

To evaluate the biological effect of *IGFBP7*, two kinds of plasmids coding for shRNA against IGFBP7 (no. 1 and no. 2) were transfected into PLC-P and named as PLC-P/shRNA no. 1 and PLC-P/shRNA no. 2. The IGFBP7 expression was suppressed at both mRNA and protein levels in the established PLC-P/shRNAs, which was confirmed by qRT-PCR and western blot analysis, respectively (Figure 2A). The MTT assay showed that PLC-P/shRNAs were significantly more resistant to IFN-*α* than PLC-P/shRNA-NC (Figure 2B). On the basis of the measurement of IC_50_, the fold increase of IC_50_ to IFN-*α* was much larger than that to other drugs, including 5-FU, CDDP, and DXR, suggesting that chemoresistance acquired by IGFBP7 is specific to IFN-*α* (Table 1). IFNAR2, STAT1, and STAT2 were similarly expressed in PLC-P/shRNA and PLC-P/shRNA-NC, and the IFN-*α*-induced pSTAT1 and pSTAT2 expressions were also not similar in the two cells (Supplementary Figure S1A).

**Figure 3:**
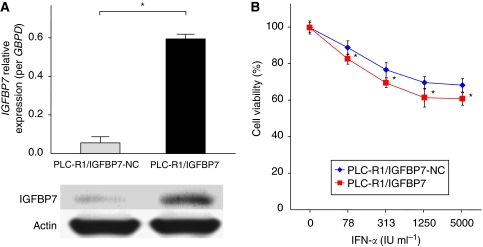
Characteristics of IFN-resistant PLC/PRF/5 cell clones (PLC-R1) transfected with *Insulin-like growth factor-binding protein 7* (*IGFBP7*) expression plasmid. (**A**) Quantitative reverse transcriptase-PCR and western blot analysis showed that the IGFBP7 expression level in PLC-R1/IGFBP7 was significantly higher than that in PLC-R1/IGFBP7-negative control (NC). (**B**) MTT assay showed that PLC-R1/IGFBP7 were significantly more sensitive to IFN-*α* than was PLC-R1/IGFBP7-NC. Data are mean±s.d. ^*^P<0.05.

As IGFBP7 has been shown to suppress tumour activity through induction of apoptosis (Landberg *et al*, 2001; Mutaguchi *et al*, 2003; Sato *et al*, 2007; Wajapeyee *et al*, 2008), we evaluated the extent of apoptosis induced at 24 h after treatment of PLC-P/shRNA with 1000 IU ml^−1^ IFN-*α*. Annexin V assay using flow cytometry showed a significantly lower percentage of early apoptotic cells in PLC-P/shRNA than in PLC-P/shRNA-NC (Figure 3C). Moreover, the activity of caspase-3, caspase-8, and caspase-9 induced by IFN-*α* in PLC-P/shRNA was significantly lower than that by PLC-P/shRNA-NC (Figure 3D). A plasmid coding for shRNA against IGFBP7 was transfected in other liver cancer cell lines (HLE and Hep3B). Both these cell lines showed downregulated *IGFBP7* expression (Supplementary Figure S2A). The transfected HLE and Hep3B cells were also resistant to IFN-*α* treatment (500 IU ml^−1^) (Supplementary Figure S2B).

As IGFBP7 has been reported to bind insulin and IGF (Oh, 1998; Subramanian *et al*, 2007), it could be conceivable that IGFBP7 induces resistance by interfering with insulin and/or IGF signalling. To verify this possibility, we examined the effect of IGFBP7 on the phosphorylation of IRS-1 and Akt, major transducers of insulin and IGF signalling. As shown in Supplementary Figure S1B, there were no significant differences in the phosphorylation of IRS-1 or Akt between PLC-P/shRNA and PLC-P/shRNA-NC. This result suggests that IGFBP7-related resistance occurs in an insulin- and IGF-independent manner.

### Transfection of IGFBP7 restores sensitivity to IFN-*α*

Next, *IGFBP7* expression plasmid was transfected into PLC-R1 (PLC-R1/IGFBP7). Upregulation of IGFBP7 in PLC-R1/IGFBP7 compared with PLC-R1/IFGBP7-NC was confirmed by qRT-PCR and western blot analysis (Figure 3A). By the MTT assay, PLC-R1/IGFBP7 partially but significantly restores sensitivity to IFN-*α* compared with PLC-R1/IGFBP7-NC (Figure 3B).

### IGFBP7 is a useful predictor of clinical response to IFN-*α*/5-FU therapy

**Figure 4:**
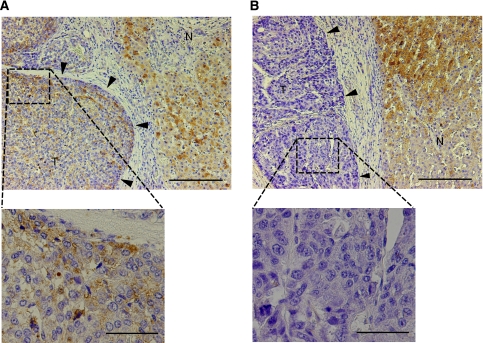
Immunohistochemistry for Insulin-like growth factor-binding protein 7 (IGFBP7) in representative hepatocellular carcinoma cases (**A**) A representative IGFBP7-positive case. The IGFBP7 expression was shown in the cytoplasm of normal liver cells and in the majority of tumour cells. (**B**) A representative IGFBP7-negative case. The IGFBP7 expression was not identified in tumour cells. Upper panel, low-power field (Bar=200 *μ*m); lower panel, high-power field (Bar=50 *μ*m); T, tumour lesion (arrowheads); N, non-tumour lesion.

To confirm whether IGFBP7 expression is associated with the clinical response to IFN-*α*/5-FU therapy, HCC samples of 30 patients who underwent IFN-*α*/5-FU therapy postoperatively were immunohistochemically stained for IGFBP7 expression. Whereas the expression levels of IGFBP7 in cancerous lesions varied among the patients, a homogenous staining for IGFBP7 was observed in the cytoplasm of cells in non-cancerous sections (Figure 4). Among the 30 patients examined, 12 (40.0%) showed positive staining, whereas 18 (60.0%) patients were negative for IGFBP7. Of the IGFBP7-positive patients, 66.7% (8 of 12) were histologically evaluated as responders to the therapy, whereas only 11.1% (2 of 18) of IGFBP7-negative patients were responders, suggesting that IGFBP7 expression was significantly associated with response to therapy (*P*<0.05) (Table 2). The sensitivity, specificity, and accuracy for the prediction to IFN-*α*/5-FU therapy by IGFBP7 were 80.0% (8 of 10), 80.0% (16 of 20), and 80.0% (24 of 30). None of the other clinicopathological factors tested, apart from IFNAR2, were associated with response to the therapy (Supplementary Table S2).

**Figure 5:**
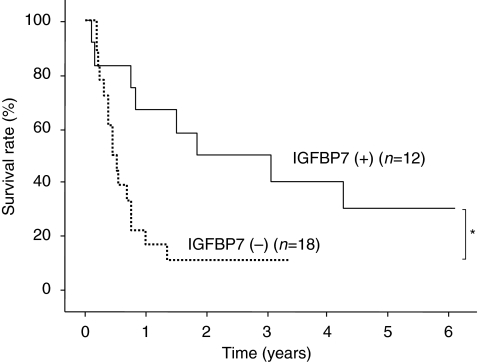
Postoperative overall survival curves showed a significantly better survival rate for Insulin-like growth factor-binding protein 7 (IGFBP7)-positive patients than for IGFBP7-negative patients (^*^P<0.05).

Finally, we examined the correlations between postoperative prognosis and various clinicopathological factors including IGFBP7 status. The postoperative overall survival in IGFBP7-positive patients was significantly better than that in IGFBP7-negative patients (*P*<0.05, Figure 5). Furthermore, multivariate analysis of overall survival using two significant factors identified in the univariate analyses showed that, in addition to IFNAR2, IGFBP7 status was an independent and significant determinant of overall survival (Table 3), indicating that IGFBP7 is a potentially useful marker for the prediction of clinical response to IFN-*α*/5-FU therapy.

## Discussion

In this study, gene expression profiling identified significant suppression of IGFBP7 in PLC-Rs compared with PLC-P. Insulin-like growth factor-binding protein 7, also known as IGFBP-rP1 and MAC25, can inhibit the proliferation of cancer cells, and its expression is downregulated in certain cancers (Burger *et al*, 1998; Landberg *et al*, 2001; Mutaguchi *et al*, 2003; Sato *et al*, 2007; Lin *et al*, 2008; Wajapeyee *et al*, 2008). It is also reported that IGFBP7 suppression is associated with rapid tumour growth and tumour invasiveness (Burger *et al*, 1998; Sato *et al*, 2007; Lin *et al*, 2008). However, there are no reports of the association between IGFBP7 expression and sensitivity to chemotherapeutic drugs.

In this study, IGFBP7 was suppressed by shRNA transfection in HCC cells and the transfected cells acquired resistance to IFN-*α*. The association between IGFBP7 expression and response to IFN-*α* was also confirmed in experiments using IGFBP7-overexpressing cells. Considering that IGFBP7-suppressed cells showed a smaller percentage of apoptosis than control cells, the acquired resistance was thought to result from the impediment of apoptosis. The suppression of apoptosis by downregulation of IGFBP7 was consistent with that found in previous studies (Burger *et al*, 1998; Landberg *et al*, 2001; Mutaguchi *et al*, 2003; Sato *et al*, 2007; Lin *et al*, 2008; Wajapeyee *et al*, 2008). In addition to resistance to IFN-*α*, IGFBP7-suppressed cells showed modest but significant resistance to other drugs. Taking into consideration the fact that IGFBP7 promotes apoptosis even in the absence of any drugs, the acquisition of resistance to both IFN-*α* and other drugs may be quite natural. However, the fold increase in acquired resistance to IFN-*α* was much larger than that to other drugs as confirmed by measurements of IC_50_, suggesting that IGFBP7 is specifically related to the resistance to IFN-*α*. Moreover, from the experiments of insulin- and IGF signalling, this effect of IGFBP7 was suggestive to occur in an insulin- and IGF-independent manner.

Furthermore, to clarify the mechanism of IGFBP7-specific IFN resistance, we examined IFNAR2 expression and IFN signalling and compared them between PLC-P and PLC-Rs and between PLC-P/shRNA and PLC-P/shRNA-NC. The IFN signalling was evaluated by the expression of STAT1 and STAT2, and by IFN-*α*-induced expression of pSTAT1 and pSTAT2. The results showed no significant differences in the expression of IFNAR2 and IFN signalling between PLC-P and PLC-Rs or between PLC-P/shRNA and PLC-P/shRNA-NC. On the other hand, Wajapeyee *et al* (2008) reported that IGFBP7 induces apoptosis through increased SMARCB1 upregulation by the recruitment of STAT1 to the binding site of the SMARCB1 promoter. Another study reported that STAT1 is recruited to the SMARCB1 promoter by IFN, suggesting that IFN-induced STAT1 recruitment to the SMARCB1 promoter is possibly one of the mechanisms of IFN-induced apoptosis (Hartman *et al*, 2005). It might therefore be possible that STAT1 recruitment could be prevented antagonistically when IGFBP7 is suppressed, leading to a higher resistance to IFN-*α* than to other drugs. In this study, however, pSTAT1 expression was not different between PLC-P and PLC-Rs or between PLC-P/shRNA and PLC-P/shRNA-NC, and there were no significant differences in the SMARCB1 expression evaluated by the result of microarray between PLC-P and PLC-Rs. These results indicate that IGFBP7-related IFN resistance is based not on SMARCB1 but on a novel mechanism, which should be clarified in the future.

The present study revealed that, in addition to the significant association between IGFBP7 status and the clinical response to IFN-*α*/5-FU therapy, the IGFBP7 status as well as IFNAR2, was an independent prognostic factor in HCC patients undergoing IFN-*α*/5-FU therapy. Because our 30 patients in this study are those with far advanced HCC, it is quite reasonable that the clinical response to the therapy correlates well with the prognosis after the therapy. These results indicate that prediction of response and prognosis by evaluating IGFBP7 and IFNAR2 is useful in this clinical setting.

In summary, IGFBP7 was selected on the basis of the results of the microarray analysis using established IFN-resistant HCC cell lines. The expression of IGFBP7 in tumour tissue correlated significantly with the response to IFN-*α*/5-FU therapy. This correlation was also confirmed in genetic manipulation experiments. Our findings suggest that IGFBP7 could be a novel marker for the prediction of the clinical response to IFN-*α*/5-FU therapy.

## Figures and Tables

**Table 1:** IC_50_ for IFN-*α*, 5-FU, CDDP, and DXR in PLC-P/shRNA-NC and PLC-P/shRNA

	**IC_50_**		
**Drug**	**PLC-P/ shRNA-NC**	**PLC-P/ shRNA**	**Fold increase (shRNA/shRNA-NC)**
IFN-*α* (IU ml^−1^)	807±96.5	11 608.0±1179.4	14.38
5-FU (*μ*g ml^−1^)	41.4±5.5	53.1±10.3	1.28
CDDP (*μ*g ml^−1^)	3.9±0.3	4.8±0.4	1.24
DXR (*μ*g ml^−1^)	1.4±0.2	2.2±0.4	1.52

Abbreviations: 5-FU=5-fluorouracil; CDDP=cisplatin; DXR=doxorubicin; IC=inhibitory concentration; IFN-*α*=interferon-*α*; NC=negative control; PLC-P=Parental PLC/PRF/5 cell; sh-RNA=short hairpin RNA.

Data are mean±s.d.

**Table 2:** Association between immunohistochemically determined IGFBP7 expression and clinical response to IFN-*α*/5-FU therapy

	**Responders**	**Non-responders**	***P*-value**
IGFBP7(+)	8	4	0.0057
IGFBP7(−)	2	16	

Abbreviations: 5-FU=5-fluorouracil; IFN-*α*=interferon-*α*; IGFBP7=insulin-like growth factor-binding protein 7.

**Table 3:** Statistical analyses of overall survival of 30 patients with advanced hepatocellular carcinoma

	**Univariate**	**Multivariate**		
	***P*-value**	**OR**	**95%CI**	***P*-value**
Age (<60/⩾60 years)	0.6846			
Gender (male/female)	0.5975			
Cirrhosis (−/+)	0.7014			
Child-Pugh classification (A/B)	0.1825			
AFP (<400/⩾400 ng ml^−1^)	0.7459			
PIVKA-II (<1000/⩾1000 mAU l^−1^)	0.6637			
Histological grade (mod, poor/undifferentiated)	0.1705			
IFNAR2 status (−/+)	0.0010	2.645	1.024–6.831	0.0056
IGFBP7 status (−/+)	0.0170	4.096	1.511–11.108	0.0445

Abbreviations: AFP=95% CI=95% confidence interval; α-fetoprotein; IFNAR2=type I interferon receptor 2; IGFBP7=insulin-like growth factor-binding protein 7; mod=moderately differentiated; OR=odds ratio; PIVKA-II=protein induced by vitamin K absence; poor=poorly differentiated.

## References

[bib1] Asahara T, Itamoto T, Katayama K, Nakahara H, Hino H, Yano M, Ono E, Dohi K, Nakanishi T, Kitamoto M, Azuma K, Itoh K, Shimamoto F (1999) Hepatic resection with tumor thrombectomy for hepatocellular carcinoma with tumor thrombi in the major vasculatures. Hepatogastroenterology 46: 1862–1869 10430360

[bib2] Burger AM, Zhang X, Li H, Ostrowski JL, Beatty B, Venanzoni M, Papas T, Seth A (1998) Down-regulation of T1A12/mac25, a novel insulin-like growth factor binding protein related gene, is associated with disease progression in breast carcinomas. Oncogene 16: 2459–2467 9627112 10.1038/sj.onc.1201772

[bib3] Chung YH, Song IH, Song BC, Lee GC, Koh MS, Yoon HK, Lee YS, Sung KB, Suh DJ (2000) Combined therapy consisting of intraarterial cisplatin infusion and systemic interferon-alpha for hepatocellular carcinoma patients with major portal vein thrombosis or distant metastasis. Cancer 88: 1986–1991 10813709

[bib4] Damdinsuren B, Nagano H, Monden M (2007a) Combined intra-arterial 5-fluorouracil and subcutaneous interferon-α therapy for highly advanced hepatocellular carcinoma. Hepatol Res 37(Suppl 2): S238–S250 17877489 10.1111/j.1872-034X.2007.00191.x

[bib5] Damdinsuren B, Nagano H, Wada H, Noda T, Natsag J, Marubashi S, Miyamoto A, Takeda Y, Umeshita K, Doki Y, Dono K, Monden M (2007b) Interferon α receptors are important for antiproliferative effect of interferon-α against human hepatocellular carcinoma cells. Hepatol Res 37: 77–83 17300701 10.1111/j.1872-034X.2007.00007.x

[bib6] Eguchi H, Nagano H, Yamamoto H, Miyamoto A, Kondo M, Dono K, Nakamori S, Umeshita K, Sakon M, Monden M (2000) Augmentation of antitumor activity of 5-fluorouracil by interferon α is associated with up-regulation of p27Kip1 in human hepatocellular carcinoma cells. Clin Cancer Res 6: 2881–2890 10914738

[bib7] Furuse J, Iwasaki M, Yoshino M, Konishi M, Kawano N, Kinoshita T, Ryu M, Satake M, Moriyama N (1997) Hepatocellular carcinoma with portal vein tumor thrombus: embolization of arterioportal shunts. Radiology 204: 787–790 9280260 10.1148/radiology.204.3.9280260

[bib8] Hartman SE, Bertone P, Nath AK, Royce TE, Gerstein M, Weissman S, Snyder M (2005) Global changes in STAT target selection and transcription regulation upon interferon treatments. Genes Dev 19: 2953–2968 16319195 10.1101/gad.1371305 PMC1315400

[bib9] Kondo M, Nagano H, Sakon M, Yamamoto H, Morimoto O, Arai I, Miyamoto A, Eguchi H, Dono K, Nakamori S, Umeshita K, Wakasa K, Ohmoto Y, Monden M (2000) Expression of interferon α/β receptor in human hepatocellular carcinoma. Int J Oncol 17: 83–88 10853022

[bib10] Kondo M, Nagano H, Wada H, Damdinsuren B, Yamamoto H, Hiraoka N, Eguchi H, Miyamoto A, Yamamoto T, Ota H, Nakamura M, Marubashi S, Dono K, Umeshita K, Nakamori S, Sakon M, Monden M (2005) Combination of IFN-α and 5-fluorouracil induces apoptosis through IFN-α/β receptor in human hepatocellular carcinoma cells. Clin Cancer Res 11: 1277–1286 15709199

[bib11] Kondo M, Yamamoto H, Nagano H, Okami J, Ito Y, Shimizu J, Eguchi H, Miyamoto A, Dono K, Umeshita K, Matsuura N, Wakasa K, Nakamori S, Sakon M, Monden M (1999) Increased expression of COX-2 in nontumor liver tissue is associated with shorter disease-free survival in patients with hepatocellular carcinoma. Clin Cancer Res 5: 4005–4012 10632332

[bib12] Landberg G, Ostlund H, Nielsen NH, Roos G, Emdin S, Burger AM, Seth A (2001) Downregulation of the potential suppressor gene IGFBP-rP1 in human breast cancer is associated with inactivation of the retinoblastoma protein, cyclin E overexpression and increased proliferation in estrogen receptor negative tumors. Oncogene 20: 3497–3505 11429696 10.1038/sj.onc.1204471

[bib13] Lee HS, Kim JS, Choi IJ, Chung JW, Park JH, Kim CY (1997) The safety and efficacy of transcatheter arterial chemoembolization in the treatment of patients with hepatocellular carcinoma and main portal vein obstruction. A prospective controlled study. Cancer 79: 2087–2094 9179054

[bib14] Leung TW, Patt YZ, Lau WY, Ho SK, Yu SC, Chan AT, Mok TS, Yeo W, Liew CT, Leung NW, Tang AM, Johnson PJ (1999) Complete pathological remission is possible with systemic combination chemotherapy for inoperable hepatocellular carcinoma. Clin Cancer Res 5: 1676–1681 10430068

[bib15] Lin J, Lai M, Huang Q, Ruan W, Ma Y, Cui J (2008) Reactivation of IGFBP7 by DNA demethylation inhibits human colon cancer cell growth *in vitro*. Cancer Biol Ther 7: 1896–1900 18981723 10.4161/cbt.7.12.6937

[bib16] Lugli E, Troiano L, Ferraresi R, Roat E, Prada N, Nasi M, Pinti M, Cooper EL, Cossarizza A (2005) Characterization of cells with different mitochondrial membrane potential during apoptosis. Cytometry A 68: 28–35 16184612 10.1002/cyto.a.20188

[bib17] Mutaguchi K, Yasumoto H, Mita K, Matsubara A, Shiina H, Igawa M, Dahiya R, Usui T (2003) Restoration of insulin-like growth factor binding protein-related protein 1 has a tumor-suppressive activity through induction of apoptosis in human prostate cancer. Cancer Res 63: 7717–7723 14633696

[bib18] Nagano H, Miyamoto A, Wada H, Ota H, Marubashi S, Takeda Y, Dono K, Umeshita K, Sakon M, Monden M (2007a) Interferon-α and 5-fluorouracil combination therapy after palliative hepatic resection in patients with advanced hepatocellular carcinoma, portal venous tumor thrombus in the major trunk, and multiple nodules. Cancer 110: 2493–2501 17941012 10.1002/cncr.23033

[bib19] Nagano H, Sakon M, Eguchi H, Kondo M, Yamamoto T, Ota H, Nakamura M, Wada H, Damdinsuren B, Marubashi S, Miyamoto A, Takeda Y, Dono K, Umeshit K, Nakamori S, Monden M (2007b) Hepatic resection followed by IFN-α and 5-FU for advanced hepatocellular carcinoma with tumor thrombus in the major portal branch. Hepatogastroenterology 54: 172–179 17419255

[bib20] Nakamura M, Nagano H, Sakon M, Yamamoto T, Ota H, Wada H, Damdinsuren B, Noda T, Marubashi S, Miyamoto A, Takeda Y, Umeshita K, Nakamori S, Dono K, Monden M (2007) Role of the Fas/FasL pathway in combination therapy with interferon-α and fluorouracil against hepatocellular carcinoma *in vitro*. J Hepatol 46: 77–88 17045692 10.1016/j.jhep.2006.07.032

[bib21] Noda T, Nagano H, Takemasa I, Yoshioka S, Murakami M, Wada H, Kobayashi S, Marubashi S, Takeda Y, Dono K, Umeshita K, Matsuura N, Matsubara K, Doki Y, Mori M, Monden M (2009) Activation of Wnt/β-catenin signalling pathway induces chemoresistance to interferon-α/5-fluorouracil combination therapy for hepatocellular carcinoma. Br J Cancer 100: 1647–1658 19401692 10.1038/sj.bjc.6605064 PMC2696759

[bib22] Obi S, Yoshida H, Toune R, Unuma T, Kanda M, Sato S, Tateishi R, Teratani T, Shiina S, Omata M (2006) Combination therapy of intraarterial 5-fluorouracil and systemic interferon-α for advanced hepatocellular carcinoma with portal venous invasion. Cancer 106: 1990–1997 16565970 10.1002/cncr.21832

[bib23] Oh Y (1998) IGF-independent regulation of breast cancer growth by IGF binding proteins. Breast Cancer Res Treat 47: 283–293 9516082 10.1023/a:1005911319432

[bib24] Oken MM, Creech RH, Tormey DC, Horton J, Davis TE, McFadden ET, Carbone PP (1982) Toxicity and response criteria of the Eastern Cooperative Oncology Group. Am J Clin Oncol 5: 649–655 7165009

[bib25] Ota H, Nagano H, Sakon M, Eguchi H, Kondo M, Yamamoto T, Nakamura M, Damdinsuren B, Wada H, Marubashi S, Miyamoto A, Dono K, Umeshita K, Nakamori S, Wakasa K, Monden M (2005) Treatment of hepatocellular carcinoma with major portal vein thrombosis by combined therapy with subcutaneous interferon-α and intra-arterial 5-fluorouracil; role of type 1 interferon receptor expression. Br J Cancer 93: 557–564 16106266 10.1038/sj.bjc.6602742 PMC2361594

[bib26] Patt YZ, Hassan MM, Lozano RD, Brown TD, Vauthey JN, Curley SA, Ellis LM (2003) Phase II trial of systemic continuous fluorouracil and subcutaneous recombinant interferon Alfa-2b for treatment of hepatocellular carcinoma. J Clin Oncol 21: 421–427 12560429 10.1200/JCO.2003.10.103

[bib27] Sakon M, Nagano H, Dono K, Nakamori S, Umeshita K, Yamada A, Kawata S, Imai Y, Iijima S, Monden M (2002) Combined intraarterial 5-fluorouracil and subcutaneous interferon-α therapy for advanced hepatocellular carcinoma with tumor thrombi in the major portal branches. Cancer 94: 435–442 11900229 10.1002/cncr.10246

[bib28] Sato Y, Chen Z, Miyazaki K (2007) Strong suppression of tumor growth by insulin-like growth factor-binding protein-related protein 1/tumor-derived cell adhesion factor/mac25. Cancer Sci 98: 1055–1063 17465992 10.1111/j.1349-7006.2007.00502.x PMC11158653

[bib29] Subramanian A, Sharma AK, Banerjee D, Jiang WG, Mokbel K (2007) Evidence for a tumour suppressive function of IGF1-binding proteins in human breast cancer. Anticancer Res 27: 3513–3518 17972510

[bib30] Tanaka A, Morimoto T, Yamaoka Y (1996) Implications of surgical treatment for advanced hepatocellular carcinoma with tumor thrombi in the portal vein. Hepatogastroenterology 43: 637–643 8799408

[bib31] Urabe T, Kaneko S, Matsushita E, Unoura M, Kobayashi K (1998) Clinical pilot study of intrahepatic arterial chemotherapy with methotrexate, 5-fluorouracil, cisplatin and subcutaneous interferon-α-2b for patients with locally advanced hepatocellular carcinoma. Oncology 55: 39–47 9428374 10.1159/000011833

[bib32] Wada H, Nagano H, Yamamoto H, Arai I, Ota H, Nakamura M, Damdinsuren B, Noda T, Marubashi S, Miyamoto A, Takeda Y, Umeshita K, Doki Y, Dono K, Nakamori S, Sakon M, Monden M (2007) Combination therapy of interferon-α and 5-fluorouracil inhibits tumor angiogenesis in human hepatocellular carcinoma cells by regulating vascular endothelial growth factor and angiopoietins. Oncol Rep 18: 801–809 17786339

[bib33] Wada H, Nagano H, Yamamoto H, Noda T, Murakami M, Kobayashi S, Marubashi S, Eguchi H, Takeda Y, Tanemura M, Umeshita K, Doki Y, Mori M (2009) Combination of interferon-alpha and 5-fluorouracil inhibits endothelial cell growth directly and by regulation of angiogenic factors released by tumor cells. BMC Cancer 9: 361 19821965 10.1186/1471-2407-9-361 PMC2767355

[bib34] Wajapeyee N, Serra RW, Zhu X, Mahalingam M, Green MR (2008) Oncogenic BRAF induces senescence and apoptosis through pathways mediated by the secreted protein IGFBP7. Cell 132: 363–374 18267069 10.1016/j.cell.2007.12.032 PMC2266096

[bib35] Yamakado K, Tanaka N, Nakatsuka A, Matsumura K, Takase K, Takeda K (1999) Clinical efficacy of portal vein stent placement in patients with hepatocellular carcinoma invading the main portal vein. J Hepatol 30: 660–668 10207808 10.1016/s0168-8278(99)80197-4

[bib36] Yamamoto T, Nagano H, Sakon M, Wada H, Eguchi H, Kondo M, Damdinsuren B, Ota H, Nakamura M, Marubashi S, Miyamoto A, Dono K, Umeshita K, Nakamori S, Yagita H, Monden M (2004) Partial contribution of tumor necrosis factor-related apoptosis-inducing ligand (TRAIL)/TRAIL receptor pathway to antitumor effects of interferon-α/5-fluorouracil against hepatocellular carcinoma. Clin Cancer Res 10: 7884–7895 15585621 10.1158/1078-0432.CCR-04-0794

