# Supplementary material for: Insulin-like growth factor-binding protein 7 alters the sensitivity to interferon-based anticancer therapy in hepatocellular carcinoma cells
Source: Br J Cancer. 2010 Apr 20;102(10):1483–90. doi: 10.1038/sj.bjc.6605669 (PMC2869168; doi:10.1038/sj.bjc.6605669)
Supplement: Supplementary Figures S1 and S2 [file 6605669x1.ppt]

## Slide 1
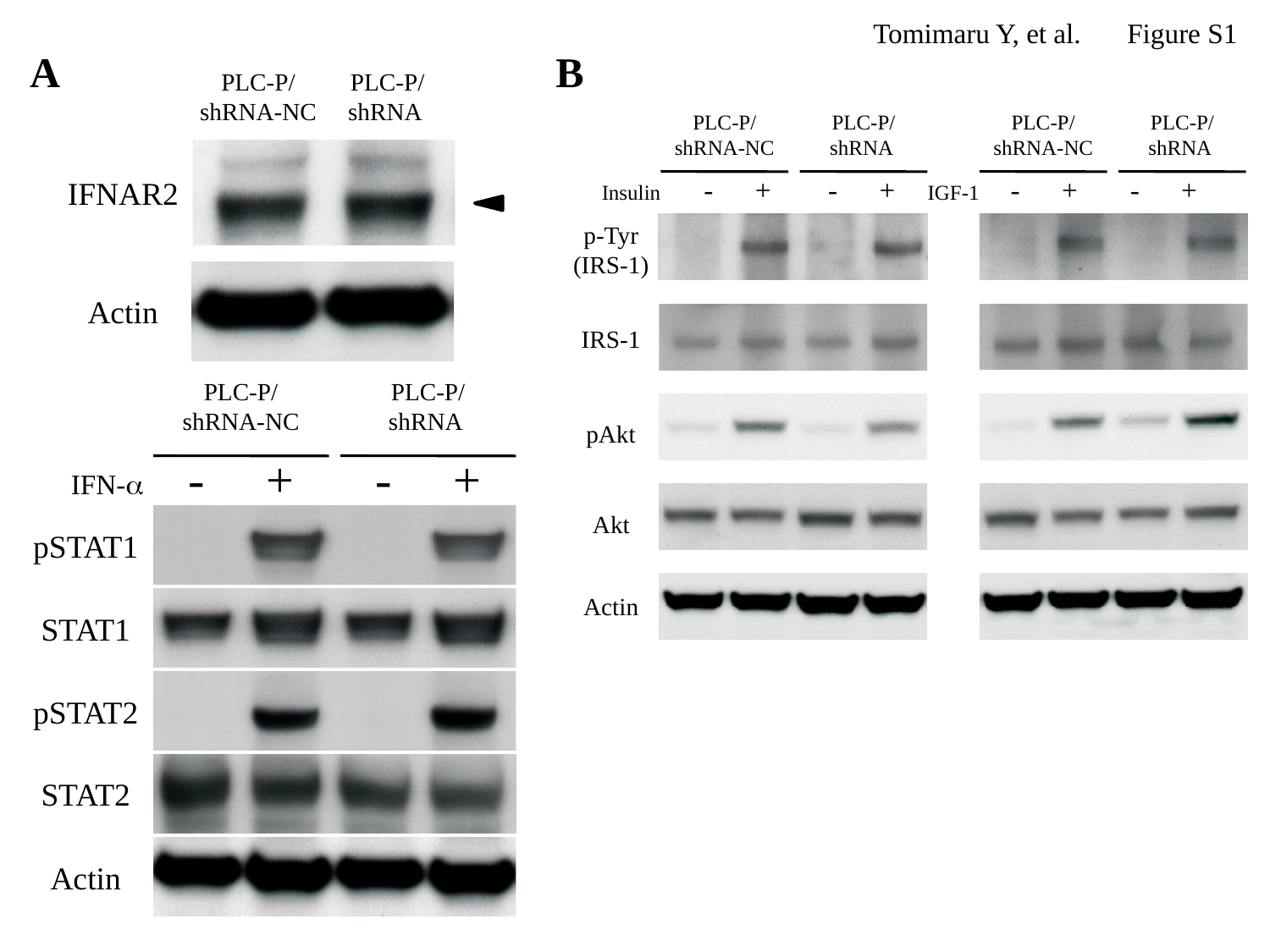

Tomimaru Y, et al.	Figure S1
A
B
PLC-P/
shRNA-NC
PLC-P/
shRNA
PLC-P/
shRNA-NC
PLC-P/
shRNA
PLC-P/
shRNA-NC
PLC-P/
shRNA
- +
- +
- +
- +
IFNAR2
Insulin
IGF-1
p-Tyr
(IRS-1)
Actin
IRS-1
PLC-P/
shRNA-NC
PLC-P/
shRNA
pAkt
- +
- +
IFN-α
Akt
pSTAT1
Actin
STAT1
pSTAT2
STAT2
Actin

## Slide 2
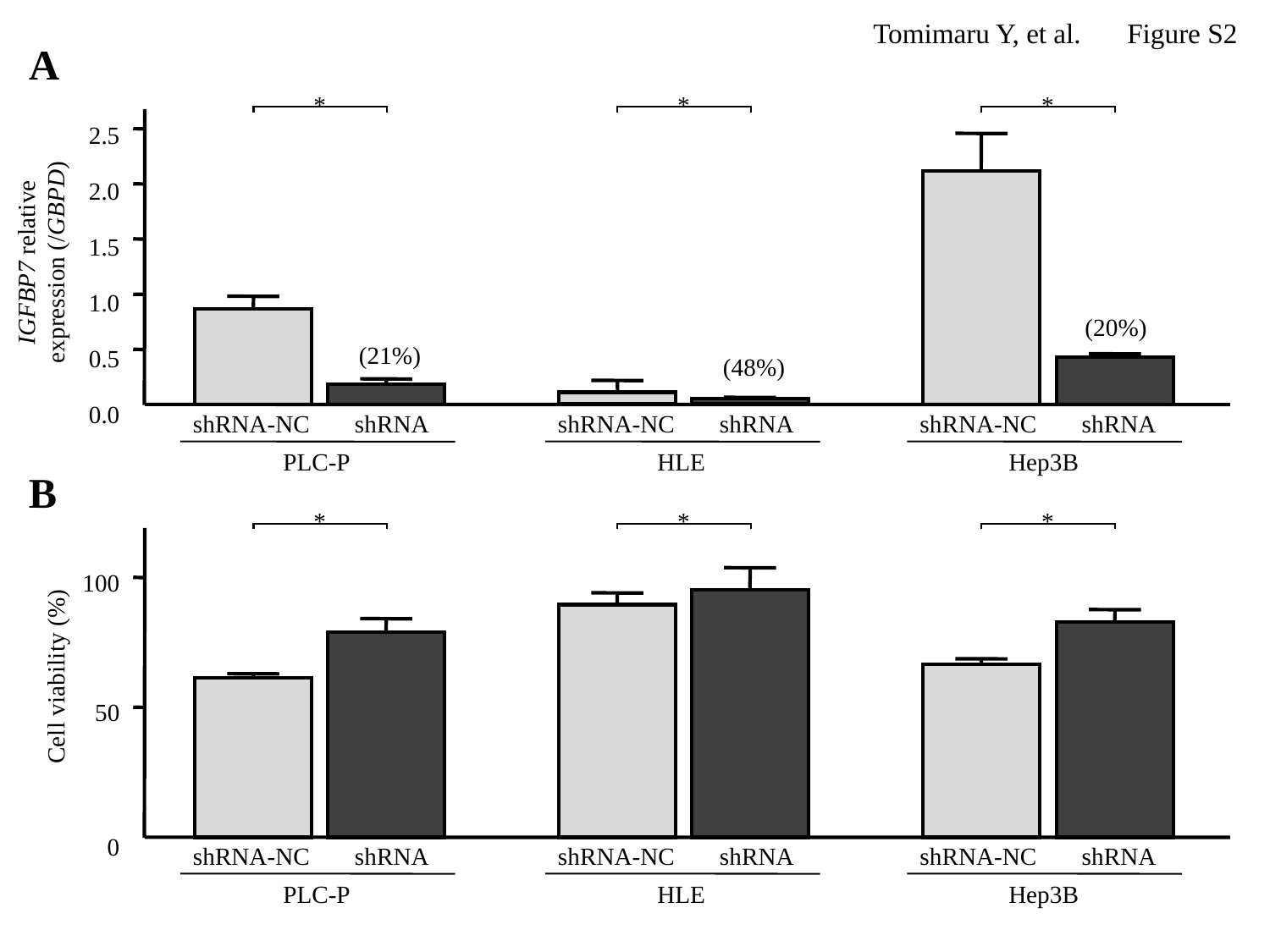

Tomimaru Y, et al.	Figure S2
A
*
*
*
2.5
2.0
1.5
1.0
(20%)
(21%)
0.5
(48%)
IGFBP7 relative
expression (/GBPD)
0.0
shRNA-NC
shRNA
shRNA-NC
shRNA
shRNA-NC
shRNA
PLC-P
HLE
Hep3B
B
*
*
*
100
Cell viability (%)
50
0
shRNA-NC
shRNA
shRNA-NC
shRNA
shRNA-NC
shRNA
PLC-P
HLE
Hep3B
