# Supplementary material for: Insulin-like growth factor-binding protein 7 alters the sensitivity to interferon-based anticancer therapy in hepatocellular carcinoma cells
Source: Br J Cancer. 2010 Apr 20;102(10):1483–90. doi: 10.1038/sj.bjc.6605669 (PMC2869168; doi:10.1038/sj.bjc.6605669)
Supplement: Supplementary Information [file 6605669x2.doc]

**SUPPLEMENTARY ONLINE MATERIALS**

Table S1. List of identified 107 genes up- and downregulated by more than 1.5-fold in IFN-resistant cells (PLC-R1, PLC-R2, and PLC-R3) with parental cells (PLC-P).

| Rank | Status | Fold change | Gene symbol | Gene name | Ref Seq ID |
| --- | --- | --- | --- | --- | --- |
| 1 | Down | 3.282 | UCHL1 | ubiquitin carboxyl-terminal esterase l1 (ubiquitin thiolesterase); uchl1 | NM_004181 |
| 2 | Down | 2.963 | IGFBP7 | insulin-like growth factor-binding protein 7; igfbp7 | NM_001553 |
| 3 | Down | 2.749 | - | ensembl genscan prediction | - |
| 4 | Up | 2.600 | - | hypothetical protein mgc3130; mgc3130 | NM_024032 |
| 5 | Down | 2.595 | - | ensembl genscan prediction | - |
| 6 | Down | 2.536 | - | hect domain and rld 2 herc2 | NM_004667 |
| 7 | Up | 2.489 | CFHL2 | H factor (complement)-like 3; hfl3 | NM_005666 |
| 8 | Up | 2.461 | - | calcitonin gene-related peptide-receptor component protein; cgrp-rcp | NM_014478 |
| 9 | Down | 2.431 | TEKT3 | testicular microtubules-related protein tektin3; tekt3 | NM_031898 |
| 10 | Up | 2.390 | - | ensembl genscan prediction | - |
| 11 | Up | 2.358 | - | hypothetical protein xp_034790; loc90900 | - |
| 12 | Up | 2.331 | ZNF343 | hypothetical protein mgc10715; mgc10715 | NM_024325 |
| 13 | Down | 2.324 | - | nectin 3; dkfzp566b0846 | NM_015480 |
| 14 | Up | 2.313 | - | ensembl genscan prediction |  |
| 15 | Up | 2.298 | - | ensembl genscan prediction | - |
| 16 | Down | 2.297 | AKR1B10 | aldo-keto reductase family 1, member b10 (aldose reductase); akr1b10 | NM_020299 |
| 17 | Down | 2.279 | CTDSPL | hya22 | NM_005808 |
| 18 | Up | 2.237 | - | hypothetical protein xp_027888; loc89984 | - |
| 19 | Up | 2.237 | - | ensembl genscan prediction | - |
| 20 | Up | 2.235 | - | ensembl genscan prediction | - |
| 21 | Up | 2.233 | - | ensembl genscan prediction | - |
| 22 | Up | 2.230 | - | hor 5'beta1 | NM_033179 |
| 23 | Up | 2.218 | - | ensembl genscan prediction | - |
| 24 | Up | 2.193 | SMARCA4 | swi/snf related, matrix associated, actin dependent regulator of chromatin, subfamily a, member 4; smarca4 | NM_003072 |
| 25 | Up | 2.191 | - | ensembl genscan prediction | - |
| 26 | Down | 2.188 | - | t-cell receptor alpha; tcra | - |
| 27 | Up | 2.185 | C16orf3 | chromosome 16 open reading frame 3; c16orf3 | NM_001214 |
| 28 | Up | 2.165 | - | t-cell receptor beta-chain | - |
| 29 | Down | 2.154 | - | ensembl genscan prediction | - |
| 30 | Up | 2.146 | - | ensembl genscan prediction | - |
| 31 | Up | 2.137 | LAMB3 | laminin subunit beta 3 precursor; lamb3 | NM_000228 |
| 32 | Up | 2.126 | - | ensembl genscan prediction | - |
| 33 | Down | 2.113 | - | ensembl genscan prediction | - |
| 34 | Up | 2.113 | FALZ | cdna: flj23531 fis clone lng06065; unnamed protein product | NM_182641 |
| 35 | Up | 2.105 | ELL | ell gene (11-19 lysine-rich leukemia gene); ell | NM_006532 |
| 36 | Up | 2.100 | MCC | mutated in colorectal cancers | NM_002387 |
| 37 | Up | 2.092 | - | ensembl genscan prediction | - |
| 38 | Up | 2.083 | - | ensembl genscan prediction | - |
| 39 | Up | 2.079 | PODXL | podocalyxin-like; podxl | NM_005397 |
| 40 | Up | 2.061 | - | hypothetical protein xp_031748; loc113426 | - |
| 41 | Up | 2.052 | - | hypothetical protein xp_071464; loc126383 | - |
| 42 | Up | 2.049 | - | hypothetical protein flj23594; flj23594 | NM_024781 |
| 43 | Up | 2.042 | - | ensembl genscan prediction | - |
| 44 | Up | 2.042 | - | ensembl genscan prediction | - |
| 45 | Up | 2.038 | - | ensembl genscan prediction | - |
| 46 | Up | 2.035 | - | ensembl genscan prediction | - |
| 47 | Up | 2.023 | - | ensembl genscan prediction | - |
| 48 | Up | 2.012 | ACVRL1 | activin a receptor type ii-like 1; acvrl1 | NM_000020 |
| 49 | Up | 2.003 | ZSWIM5 | kiaa1511 protein; kiaa1511 | NM_020883 |
| 50 | Up | 1.983 | P2RY4 | pyrimidinergic receptor p2y, g-protein coupled, 4; p2ry4 | NM_002565 |
| 51 | Up | 1.974 | - | ensembl genscan prediction | - |
| 52 | Up | 1.967 | HSD11B1 | hydroxysteroid (11-beta) dehydrogenase 1; hsd11b1 | NM_181755 |
| 53 | Up | 1.964 | TXNDC3 | nm23-h8; loc51314 | NM_016616 |
| 54 | Up | 1.944 | CTSS | cathepsin s; ctss | NM_004079 |
| 55 | Up | 1.932 | PKD2L2 | polycystic kidney disease 2-like 2; pkd2l2 | NM_014386 |
| 56 | Up | 1.914 | - | hypothetical protein xp_029164; akap9 | - |
| 57 | Up | 1.899 | KCNAB2 | potassium voltage-gated channel, shaker-related subfamily, beta member 2; kcnab2 | NM_172130 |
| 58 | Up | 1.899 | IL17F | interleukin-17f; il17f | NM_052872 |
| 59 | Up | 1.899 | - | pro0246 protein; pro0246 | - |
| 60 | Up | 1.898 | ZNF552 | hypothetical protein flj21603; flj21603 | NM_024762 |
| 61 | Up | 1.894 | IL3 | interleukin 3 (colony-stimulating factor, multiple); il3 | NM_000588 |
| 62 | Up | 1.889 | - | hypothetical protein xp_032139; loc90490 | - |
| 63 | Up | 1.889 | - | ensembl genscan prediction | - |
| 64 | Up | 1.887 | NRP2 | neuropilin 2 |  |
| 65 | Down | 1.875 | CACNA2D1 | calcium channel, voltage-dependent, alpha 2/delta subunit 1; cacna2d1 | NM_000722 |
| 66 | Up | 1.874 | - | ensembl genscan prediction | - |
| 67 | Up | 1.873 | - | ensembl genscan prediction | - |
| 68 | Up | 1.870 | - | ensembl genscan prediction | - |
| 69 | Up | 1.863 | - | unknown (protein for mgc:13198) | NM_145039 |
| 70 | Up | 1.857 | - | ensembl genscan prediction | - |
| 71 | Down | 1.855 | - | cdna flj20582 fis clone kat12156; unnamed protein product | - |
| 72 | Up | 1.854 | TAS2R9 | taste receptor, type 2, member 9; tas2r9 | NM_023917 |
| 73 | Up | 1.836 | STRN | striatin, calmodulin binding protein; strn | NM_003162 |
| 74 | Up | 1.828 | - | ensembl genscan prediction | - |
| 75 | Up | 1.828 | - | ensembl genscan prediction | - |
| 76 | Up | 1.825 | - | ensembl genscan prediction | - |
| 77 | Up | 1.823 | - | ensembl genscan prediction | - |
| 78 | Up | 1.822 | - | hypothetical protein xp_028919; loc90096 | - |
| 79 | Up | 1.817 | MMRN2 | hypothetical protein flj13465; flj13465 | NM_024756 |
| 80 | Up | 1.813 | CLIC3 | chloride intracellular channel 3; clic3 | NM_004669 |
| 81 | Up | 1.810 | - | ensembl genscan prediction | - |
| 82 | Up | 1.801 | - | ensembl genscan prediction | - |
| 83 | Up | 1.790 | - | kiaa1321 protein; kiaa1321 | NM_020772 |
| 84 | Up | 1.786 | CFH | H factor 1 (complement); hf1 | NM_000186 |
| 85 | Up | 1.785 | - | pro0457 protein; pro0457 | - |
| 86 | Up | 1.752 | - | ensembl genscan prediction | - |
| 87 | Up | 1.749 | SLC22A1 | solute carrier family 22 (organic cation transporter), member 1; slc22a1 | NM_153187 |
| 88 | Up | 1.746 | - | ensembl prediction | - |
| 89 | Up | 1.743 | PLK4 | serine/threonine kinase 18; stk18 | NM_014264 |
| 90 | Up | 1.742 | - | ensembl genscan prediction | - |
| 91 | Up | 1.733 | SPAG11 | he2 gamma1 | NM_058202 |
| 92 | Up | 1.719 | - | ensembl genscan prediction | - |
| 93 | Up | 1.706 | ECEL1 | endothelin converting enzyme-like 1; ecel1 | NM_004826 |
| 94 | Up | 1.687 | - | ensembl genscan prediction | - |
| 95 | Up | 1.686 | - | dkfzp434a236 protein; dkfzp434a236 | NM_015512 |
| 96 | Up | 1.684 | - | immunoglobulin g heavy chain | - |
| 97 | Up | 1.673 | - | ensembl genscan prediction | - |
| 98 | Up | 1.672 | - | putative bpes syndrome breakpoint region protein | - |
| 99 | Up | 1.669 | - | ensembl genscan prediction | - |
| 100 | Up | 1.663 | GBP3 | cdna flj10961 fis clone place1000588 highly similar to interferon-induced guanylate-binding protein 1; unnamed product | NM_018284 |
| 101 | Up | 1.638 | - | ensembl genscan prediction | - |
| 102 | Up | 1.637 | DES | desmin; des | NM_001927 |
| 103 | Down | 1.635 | - | ensembl genscan prediction | - |
| 104 | Up | 1.618 | - | hypothetical protein xp_034323; loc94450 | - |
| 105 | Up | 1.591 | - | ensembl genscan prediction | - |
| 106 | Up | 1.586 | - | ensembl prediction | - |
| 107 | Up | 1.582 | OCRL | phosphatidylinositol polyphosphate 5-phosphatase, isoform a; ocrl | NM_000276 |

Status was defined as expression in PLC-Rs compared to PLC-P.

Table S2. Clinicopathological characteristics of responders and nonresponders.

|  | Responders | Non-responders | *p*-value |
| --- | --- | --- | --- |
|  | (n=10) | (n=20) |  |
| Age (years) | 59.6±8.1 | 54.4±12.7 | 0.2505 |
| Gender (male/female) | 9/1 | 2/18 | >0.9999 |
| Cirrhosis (+/-) | 4/6 | 10/10 | 0.8971 |
| Child-Pugh classification (A/B) | 7/3 | 12/8 | 0.7030 |
| AFP (ng/ml) (<400/≥400) | 5/5 | 6/14 | 0.5030 |
| PIVKA-II (mAU/l) (<1000/≥1000) | 1/9 | 5/15 | 0.6283 |
| Histological grade (mod/poor/undifferentiated) | 1/9/0 | 0/17/3 | 0.1721 |
| IFNAR2 status (-/+) | 0/10 | 10/10 | 0.0199 |

Data are mean±SD. AFP; a-fetoprotein, PIVKA-II; protein induced by vitamin K absence; mod; moderately differentiated, poor; poorly differentiated, IFNAR2, type I interferon receptor 2.

Figure S1. (A) Western blot analysis revealed similar expression levels of IFNAR2, STAT1, STAT2, pSTAT1, and pSTAT2 in PLC-P/shRNA and PLC-P/shRNA-NC. (B) Immunoprecipitation and western blot analysis showed no significant differences in the phosphorylation of IRS-1 or Akt between PLC-P/shRNA and PLC-P/shRNA-NC.

Figure S2. Characteristics of other liver cancer cells transfected with shRNA. (A) qRT-PCR confirmed the suppression of *IGFBP*7 in HLE and Hep3B transfected with shRNA against *IGFBP7* as well as PLC-P. (B) MTT assay showed that the transfected cells were more resistant to 500 IU/ml of IFN-α than non-transfected cells. Data are mean±SD. **p*<0.05.
